# One Lignanoid Compound and Four Triterpenoid Compounds with Anti-Inflammatory Activity from the Leaves of *Elaeagnus oldhamii* Maxim.

**DOI:** 10.3390/molecules181113218

**Published:** 2013-10-25

**Authors:** Chi-Ren Liao, Yu-Ling Ho, Guan-Jhong Huang, Chang Syun Yang, Che-Yi Chao, Yuan-Shiun Chang, Yueh-Hsiung Kuo

**Affiliations:** 1 School of Chinese Pharmaceutical Sciences and Chinese Medicine Resources, College of Pharmacy, China Medical University, Taichung 40402, Taiwan; 2 Nursing Department, Hung kuang University, Taichung 43302, Taiwan; 3 Health and Nutrition Biotechnology Department, Asia University, Taichung 41354, Taiwan; 4 Biotechnology Department, Asia University, Taichung 41354, Taiwan

**Keywords:** traditional herbal medicine, *Elaeagnus oldhamii* Maxim., lignanoid, triterpenoid, anti-inflammation

## Abstract

One lignanoid compound, isoamericanol B (**1**), along with four triterpenoid compounds—*cis*-3-*O*-*p*-hydroxycinnamoyloleanolic acid (**2**), *trans*-3-*O*-*p*-hydroxy cinnamoyloleanolic acid (**3**), *cis*-3-*O*-*p*-hydroxycinnamoylursolic acid (**4**), *trans*-3-*O*-*p*-hydroxycinnamoylursolic acid (**5**) have been isolated for the first time from the leaves of *Elaeagnus oldhamii* Maxim. Compounds **1**–**4** significantly inhibited the expression of NO (nitric oxide) produced in lipopolysaccharide (LPS)-stimulated RAW 264.7 cells. The IC_50_ value for inhibition of nitrite production of compound **1** was about 10.3 ± 0.4 μg/mL. In the cell viability test, however, among compounds **1**–**4** compound **1** did not significantly change cell viability. Therefore, in this study compound **1** possessed anti-inflammatory effects. The result suggests compound **1** as a potential lead compound for the treatment of inflammatory diseases.

## 1. Introduction

There are about 90 species of *Elaeagnus* around the World. The majority are native to the temperate and subtropical regions in Asia, of which nine species can be found in Taiwan [1]. Many species of *Elaeagnus* are considered as folk medicinal plants, e.g., *E. umbellate* [2], *E. pungens* [3], *E. angustifolia* [4,5] and *E. multiflora* [6]. Triterpenoids, steroids and flavonoids have been isolated from several species of *Elaeagnus*, e.g., *E. ungens* [7], *E. umbellate* [7], *E. bockii* Diels [8], *E. orientalis* [9] and *E.* pungens [10,11].

**Figure 1:**
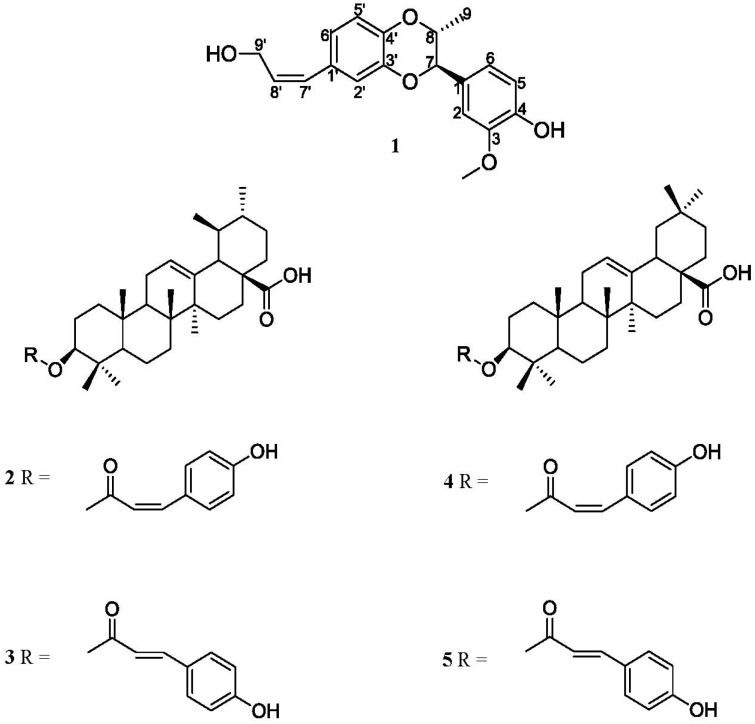
The chemical structures of compounds **1**–**5**.

*Elaeagnus oldhamii* Maxim. is a traditional herbal medicine mainly used in Taiwan to treat rheumatoid arthritis. We have investigated the analgesic and anti-inflammatory effects of the methanol extract of *E. oldhamii* Maxim. in a previous study [12]. In connection with our interest in the chemical components of this plant, in this study the EtOAc-soluble fraction has been isolated. In this study one lignanoid compound isoamericanol B (**1**) (Figure 1), as well as four triterpenoid compounds—*cis*-3-*O*-*p*-hydroxycinnamoyloleanolic acid (**2**), *trans*-3-*O*-*p*-hydroxy cinnamoyloleanolic acid (**3**), *cis*-3-*O*-*p*-hydroxycinnamoylursolic acid (**4**) and *trans*-3-*O*-*p*-hydroxycinnamoyl-ursolic acid (**5**) (Figure 1) have been isolated for the first time from the leaves of *Elaeagnus oldhamii* Maxim. The isolation and detailed structural elucidation of compound **1** and the anti-inflammatory activity of the five isolates are described herein.

## 2. Results and Discussion

The lignanoid compound isoamericanol B (**1**) has been isolated from *Elaeagnus lanceolata* Warb. ex Diels [13]. Even though this compound has been isolated in previous study, we provide here more detailed NMR spectrum information to elucidate the structure of isoamericanol B (**1**) more clearly and completely in this study.

Isoamericanol B (**1**), [α]^23^_ D_ +32.1° (*c* 0.2, MeOH), was isolated as a yellowish oil. Its molecular formula was assigned as C_19_H_20_O_5_ on the basis of the HR-ESI-MS pseudo molecular peak at *m*/*z* 327.1226 [M−H]^−^ (calc. mass for 327.1232). The IR spectrum showed an OH band (3,406 cm^−1^), and phenyl and olefin groups (3,078, 1,626, 1,610, 1,560, 1,518 and 1,506 cm^−1^). The UV absorption at λ_max_ 258.7 nm indicated a conjugated double band and an aromatic group.

The ^13^C-NMR spectrum showed the signals for 19 carbons (Table 1). After deducting one methoxyl carbon from the total of 19 carbons, the remaining 18 carbons consisted of twelve aromatic carbons, two olefinic carbons, and four sp^3^ carbons to form the structure of lignanoid compound. 

**Table 1:** NMR data (CD_3_COCD_3_) of **1**. δ in ppm, *J* in Hz.

Position	δ_H_	δ_C_
1	-	129.39
2	7.06 (*d*, *J* = 1.6, 1H)	111.30
3	-	148.50
4	-	147.57
5	6.87 (*d*, *J* = 8.2, 1H)	117.92
6	6.92 (*dd*, *J* = 8.2, 1.6, 1H)	120.30
7	5.18 (*d*, *J* = 2.6, 1H)	78.13
8	4.60 (*qd*, *J* = 6.6, 2.6, 1H)	73.81
9	1.08 (*d*, *J* = 6.6, 3H)	13.71
Phenyl-OMe	3.83 (*s*, 3H)	56.48
Phenyl-OH	7.69 (*s*, H)	-
1′	-	132.77
2′	6.87 (*d*, *J* = 1.8, 1H)	118.25
3′	-	143.94
4′	-	142.57
5′	6.86 (*d*, *J* = 8.2, 1H)	115.92
6′	6.79 (*dd*, *J* = 8.2, 1.8, 1H)	123.45
7′	6.38 (*d*, *J* = 11.8,1H)	129.64
8′	5.76 (*m*, 1H)	131.54
9′	4.38 (*d*, *J* = 5.0, 2H)	59.83
OH	3.89 (br *s*, H)	-

**Figure 2:**
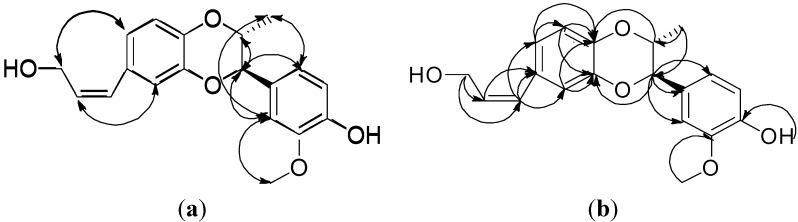
Key NOESY (↔) (**a**) correlations and HMBC connectivities (→) (**b**) of compound **1**.

^1^H-NMR spectral signals at δ 6.38 (1H, d, *J* = 11.8 Hz), δ 5.76, (1H. m), δ 4.38 (2H, d, *J* = 5.0 Hz), δ 3.89 (OH, exchangeable to D_2_O) and three ^13^C-NMR signals at δ 129.64, δ 131.54 and δ 59.83 consisted were observed. The COSY spectrum showed that signal of δ 5.76 has correlations with δ 6.38 and δ 4.38, and the HMBKC spectrum (Figure 2) showed the signal at δ 4.38 has a correlation with δ 131.54 (C-8′) and δ 129.64 (C-7′). The evidence confirmed the presence of a 1-hydroxy-2-propenyl moiety which is one of the C_3_ units attached at one of the aromatic rings. 

One set of ABX system ^1^H-NMR signals at δ 6.87 (1H, d, *J* = 1.8 Hz, H-2′), δ 6.86 (1H, d, *J* = 8.2 Hz, H-5′), δ 6.79 (1H, dd, *J* = 8.2, 1.8 Hz, H-6′) were were revealed from COSY correlation. ^13^C-NMR signals at δ 118.25, δ 115.92 and δ 123.45 were assigned as C-2′, C-5′ and C-6′ due to the corresponding HMQC correlation, and C-1′ was confirmed at δ 132.77 from the HMBC correlation with H-7′ (δ 6.38) and H-8′ (δ 5.76). The NOESY correlation (Figure 2) (H-7′/H-2′, H-6′; H-9′/H-2′, H-6′) was further proof of the location of 1-hydroxy-2-propenyl moiety at the C-1′ position. HMBC correlations between H-2′ to C-3′ (δ 143.94), C-4′ (δ 142.57), between H-5′ to C-3′ (δ 143.94), C-4′ (δ 142.57) and between H-6′ to C-5′ (δ 115.92), C-4′ (δ 142.57) were also observed. Based on the above evidence, one of monolignan units was proved to be *cis*-cinnammyl alcohol with C-3′ (δ 143.94), and C-4′ (δ 142.57) showing an *ortho*-dioxygenation pattern.

The aromatic ring on another monolignol contained a set of ABX coupling systems and an *ortho*-dioxygenated function which was revealed from following NMR spectral signals: two aromatic carbons at δ148.50 and δ147.57 (lower field than δ 150) were judged as *ortho*-dioxygenated aromatic carbons. A methoxy group (δ_H_ 3.83, 3H, s) and a phenolic hydroxy group (δ_H_ 7.69, 1H, s, exchangeable with D_2_O) attached at δ 148.50 and δ 147.57, respectively were proven by HMBC correlations. Three phenyl protons present at δ_H_ 7.06 (1H, d, *J* = 1.6 Hz), 6.87 (1H, d, *J* = 8.2 Hz) and 6.92 (1H, dd, *J* = 8.2, 1.6 Hz) resonated at δ_C_ 111.30, 117.92, and 120.30, respectively, from the HMQC experiments, and were also seen by COSY correlations.

The remaining C-4 aromatic carbon at δ 129.39 showed it was substituted with an alkyl group. The δ_H_ 7.06 signal was assigned at C-2 which was located between the methoxyl and alkyl groups due to the NOSEY correlation with the methoxy group and a *meta*-coupling constant (*J* = 1.6 Hz) with H-5. δ_H_ 6.92 with doublet of doublet coupling constant (*J* = 8.2, 1.6 Hz), and δ_H_ 6.87 with a doublet coupling constant (*J* = 8.2 Hz) can be assigned to the two protons detected at C-6 and C-5, respectively. The δ_H_ 6.87 signal at higher field than the other two phenyl protons indicated this phenyl proton was *ortho* to a phenolic hydroxyl group. Three remaining sp^3^ ^13^C-NMR signals of a propyl group presented at δ_C_ 78.13 (C-7), 73.81 (C-8), and 13.71 (C-9), and C-7 and C-8 were proposed to connect with an oxygen atom due to their lower field shift. There corresponding protons at δ_H_ at 5.18 (1H. d, *J* = 2.6 Hz, H-7), δ_H_ 4.60 (1H, qd, *J* = 6.6, 2.6 Hz, H-8) and 1.08 (3H, d, *J* = 6.6 Hz, H-9) were judged by the HMQC correlation. The COSY spectrum clarified the contiguous sequence. The signal at δ_H_ 5.18 (H-7) exhibited a HMBC correlation to C-1 (δ_C_ 129.39), C-2 (δ_C_ 111.30), and C-6 (δ_C_ 120.30). From the abovementioned evidence, the propyl residue was proposed to be linked at the *para*- position with respect to the hydroxyl group. In addition, the correlation between H-8 to C-4′ and H-7 to C-3′ allowed us to conclude that compound **1** is a 1,4-dioxane-type (8.O.4′7.O.3′) lignan.

As for the relative stereochemistry of isoamericanol B (**1**), it was judged as a *cis*-configuration based on the following evidence: The small coupling constant (*J* = 2.6 Hz) between H-7 and H-8 may be assigned as diequatorial or one axial and one equatorial. If H-7 and H-8 were positioned with a diequatorial orientation, it would be a *trans*-orientation and the methyl group and aromatic group will be in a diaxial position. In this situation. H_3_-9 would not give any NOESY correlation with the phenyl protons, but H-6 and H-2 exhibit NOESY correlations to H_3_-9, therefore, the *cis*-configuration of **1 **was unambiguously confirmed. Therefore, the structure of isoamericanol B (**1**) was elucidated as shown in Figure 1.

The ^1^H- and ^13^C-NMR spectra of the four triterpenoid compounds **2**–**5**, including *cis*-3-*O*-*p*-hydroxycinnamoyloleanolic acid [14], *trans*-3-*O*-*p*-hydroxycinnamoyl-oleanolic acid [15], *cis*-3-*O*-*p*-hydroxycinnamoylursolic acid [16] and *trans*-3-*O*-*p*-hydroxycinnamoylursolic acid [17] were compared with the spectral data reported in the literature, thus confirming their structures.

**Table 2:** Cell viability and effect of compounds **1**–**5** on LPS-induced NO production in macrophages ^a^.

Compound	Dose (μg/mL)	Cell Viability (% of Control)	No Level	NO Inhibition (% of Control)	IC_50 _(μg/mL)
Control	(−)	98.2 ± 4.4	−0.1 ± 0.3	±	
LPS	(+)	100.5 ± 4.0	30.3 ± 2.2 ^###^	±	
1	2.5	100.6 ± 5.9	17.6 ± 0.5 ^***^	42.0 ± 1.6	
	5	97.6 ± 4.3	17.5 ± 08 ^***^	42.3 ± 2.6	
	10	90.7 ± 3.3	15.2 ± 0.4 ^***^	49.7 ± 1.4	10.3 ± 0.4
	20	88.8 ± 2.6	12.0 ± 0.4 ^***^	60.4 ± 1.3	
2	2.5	87.2 ± 2.0	19.7 ± 1.4 ^**^	35.1 ± 4.6	
	5	77.9 ± 5.0	(−)	(−)	
	10	43.4 ± 1.6	(−)	(−)	
	20	28.0 ± 2.9	(−)	(−)	
3	2.5	87.6 ± 5.6	23.5 ± 1.4 ^**^	22.5 ± 4.6	
	5	86.6 ± 4.2	21.0 ± 0.7 ^***^	30.7 ± 2.2	
	10	74.1 ± 3.9	(−)	(−)	
	20	49.7 ± 8.2	(−)	(−)	
4	2.5	90.8 ± 5.4	23.0 ± 2.8 ^***^	24.2 ± 9.1	
	5	87.4 ± 3.1	20.8 ± 1.8 ^***^	31.3 ± 5.8	
	10	70.3 ± 3.5	(−)	(−)	
	20	47.6 ± 9.7	(−)	(−)	
5	2.5	93.0 ± 8.6	20.5 ± 2.6 ^***^	32.3 ± 8.6	
	5	88.8 ± 5.7	19.5 ± 1.8 ^***^	35.6 ± 6.1	
	10	88.5 ± 7.4	18.4 ± 0.8 ^***^	39.3 ± 2.6	>20
	20	86.5 ± 6.1	17.2 ± 0.9 ^***^	43.0 ± 3.1	

^a^ The data were presented as mean ± S.D. for three different experiments performed in triplicate; ### compared with sample of control group; ** *p* < 0.01, and *** *p* < 0.001 were compared with LPS-alone group.

Macrophages play an important role in the host defense system. Bacterial lipopolysaccharide (LPS) could stimulate macrophages to secret inducible nitric oxide synthases (iNOS), NO (nitric oxide) production and the release of pro-inflammatory mediators further [18]. On the other hands, NO has the critical role of regulating pro-inflammatory release during inflammatory processes [19]. In this study, the anti-inflammatory activity the compounds **1** to **5** isolated from *E. oldhamii* Maxim. was tested. Of these compounds **1** was estimated to decrease nitrate of LPS-stimulated production in RAW264.7 cells with an IC50 value of 10.3 ± 0.4 μg/mL. Cell viability was also evaluated (Table 2).

## 3. Experimental

### 3.1. General

UV spectra were obtained with a Shimadzu Pharmaspec-1700 (Taichung, Taiwan) UV-Visible spectrophotometer. Optical rotations were obtained with a Jasco P-1020 (Taichung, Taiwan) polarimeter. Infrared spectra were obtained with a Shimadzu IR prestige-21 Fourier transform infrared spectrophotometer. 1D- and 2D-NMR spectra were recorded with a Bruker DRX-500 FT-NMR (Taichung, Taiwan) spectrometer. Mass spectrometric (HREIMS) data were generated at the Mass Spectrometry Laboratory of the Chung Hsing University (Taichung, Taiwan). Column chromatography was performed using LiChroCART Si gel (5 μM; Taichung, Taiwan), and TLC analysis was carried out using aluminum pre-coated Si plates and the spots were visualized using a UV lamp at λ = 254 nm.

### 3.2. Collection, Extraction and Isolation

*E. oldhamii* Maxim. was collected from Jin-Shun Chen herbal garden (Nantou, Taiwan) as described in *Flora of Taiwan* [1]. A plant specimen has been deposited in the School of Chinese Pharmaceutical Sciences and Chinese Medicine Resources. The materials were totally dried in air under dark. Dried leaves of *E. oldhamii* Maxim. (10.0 kg) were cut into small pieces and soaked in methanol (70 L, 7 days × 3). After filtration, the combined extract was concentrated under reduced pressure to give a dried extract (801.0 g). The dried extract was suspended in H_2_O (2 L) and extracted with ethyl acetate (2 L, 5 times). The resulting ethyl acetate extract was concentrated to yield 302.5 g of an brown-green thick oil that was purified by 2.2 kg silica gel with particle size 0.063–0.200 mm and internal diameter of column 10 cm packed height 50 cm chromatography with using a gradient of increasing polarity with total *n*-hexane to total ethyl acetate as mobile phase and separated into 20 fractions on the basis of TLC analysis for random isolation of compounds. Fraction 14 (11.40 g) was re-separated by Sephadex LH 20 column chromatography (chloroform–methanol = 3:7), silica gel column chromatography (*n*-hexane:acetone = 1:1) and semi-preparative normal phase HPLC (*n*-hexane:ethyl acetate = 1:1) to afford pure compound **1** (11.8 mg). Fraction 8 (3.85 g) was re-separated by silica gel column chromatography (*n*-hexane-ethyl acetate = 8:2) and semi-preparative HPLC (*n*-hexane:acetone = 8:2) to afford pure compounds **2** (22.1 mg), **3** (12.5 mg), **4** (52.0 mg) and **5** (5.3 mg).

### 3.3. Isoamericanol B (**1**)

Yellowish oil; {[α]^23^_D_ +32.1° (*c* 0.2, MeOH)}; HR-ESI-MS *m/z:* 327.1226 [M−H]^−^ (calcd. for C_19_H_20_O_5_, 327.1232), UV (MeOH) λmax (log ε ):258.7 (4.13) nm. IR (KBr) ν_max_: hydroxyl band (3,406 cm^−1^), phenyl and olefinic groups (3,078, 1,626, 1,610, 1,560, 1,518 and 1,506 cm^−1^). ^1^H-NMR and ^13^C-NMR (500/125 MH_Z_, in CD_3_COCD_3_) were shown on Table 1.

### 3.4. Chemicals

The solvent used to open column isolation (Sephadex LH 20 and silica gel column) in the study, such as *n*-hexane, chloroform, ethyl acetate, acetone and methanol were all ACS grade. The HPLC grade *n*-hexane, ethyl acetate and acetone for HPLC isolation and the deuteriated solvent, acetone-*d*_6_, for NMR measurement were purchased from the Merck branch in Taipei, Taiwan. LPS (endotoxin from *Escherichia coli*, serotype 0127:B8), Carr (type IV), indomethacin, MTT (3-[4,5-dimethylthiazol-2-yl]-2,5-diphenyltetrazolium bromide) and other chemicals were purchased from Sigma Chemical Co. (St. Louis, MO, USA).

### 3.5. Cell Culture

A murine macrophage cell line RAW264.7 (BCRC No. 60001) was purchased from the Bioresources Collection and Research Center (BCRC, Hsinchu, Taiwan) of the Food Industry Research and Development Institute (Hsinchu, Taiwan). Cells were cultured in plastic dishes containing Dulbecco’s Modified Eagle Medium (DMEM, Sigma) supplemented with 10% fetal bovine serum (FBS, Sigma) in a CO_2_ incubator (5% CO_2_ in air) at 37 °C and subcultured every 3 days at a dilution of 1:5 using 0.05% trypsin-0.02% EDTA in Ca^2+^-, Mg^2+^-free phosphate-buffered saline (DPBS).

### 3.6. Cell Viability

Cells (2 × 10^5^) were cultured in 96-well plate containing DMEM supplemented with 10% FBS for 1 day to become nearly confluent. Then cells were cultured with compounds **1**–**5** in the presence of 100 ng/mL LPS (lipopolysaccharide) for 24 h. After that, the cells were washed twice with DPBS and incubated with 100 μL of 0.5 mg/mL MTT for 2 h at 37 °C testing for cell viability. The medium was then discarded and 100 μL dimethyl sulfoxide (DMSO) was added. After 30-min incubation, absorbance at 570 nm was read using a microplate reader (Molecular Devices, Sunnyvale, CA, USA).

### 3.7. Measurement of Nitric Oxide/Nitrite

NO production was indirectly assessed by measuring the nitrite levels in the cultured media and serum determined by a colorimetric method based on the Griess reaction. The cells were incubated with different concentration of samples in the presence of LPS (100 ng/mL) at 37 °C for 24 h. Then, cells were dispensed into 96-well plates, and 100 μL of each supernatant was mixed with the same volume of Griess reagent (1% sulfanilamide, 0.1% naphthylethylenediamine dihydrochloride and 5% phosphoric acid) and incubated at room temperature for 10 min, the absorbance was measured at 540 nm with a Micro-Reader (Molecular Devices). By using sodium nitrite to generate a standard curve, the concentration of nitrite was measured form absorbance at 540 nm.

### 3.8. Statistical Analysis

All data of IC50 values in each concentration (5, 10, 15 and 20 μg/mL) were expressed as mean ± SD (n = 4). IC50 values were estimated using a non-linear regression algorithm (Sigma Plot 12.0; SPSS Inc. Chicago, IL, USA). Statistical evaluation was carried out by one-way ANOVA followed by Scheffe’s multiple range tests.

## 4. Conclusions

The compound isoamericanol B (1), a lignanoid compound, which has been isolated for the first time from the leaves of *Elaeagnus oldhamii* Maxim. showed an excellent anti-inflammatory activity by decreasing nitrate of LPS-stimulated production in RAW264.7 cell with IC50 values of 10.3 ± 0.4 μg/mL. Compounds **2**–**4** also displayed an anti-inflammatory activity by inhibiting NO production. As to anti-inflammatory activity, compound **2** is stronger than compounds **3** and **4** is stronger than compound 5. The differences in anti-inflammatory activity may result from the *cis*- or *trans*- forms of the 3-*O*-*p*-hydroxycinnamoyl side chains of the oleanolic acid (compounds **2** and **3**) and ursolic acid (compounds **4** and **5**) structures. However, in the cell viability test, compound **1** is the only one that did not change significantly cell viability at the tested concentrations (2.5 to 20 μg/mL) among the compounds **1** to **4**. Therefore, for the anti-inflammatory effect and safety reasons, compound **1** may be a useful lead for the development of novel non-steroidal anti-inflammatory drugs.
